# Comparison of polyester (PET) capillary-channeled polymer (C-CP) fiber spin-down tips and size exclusion chromatography (SEC) for the isolation of HEK 293-derived extracellular vesicles (EVs)

**DOI:** 10.1007/s00216-026-06653-5

**Published:** 2026-07-09

**Authors:** Carolina Mata, Christopher D. Topper, R. Kenneth Marcus

**Affiliations:** Department of Chemistry, Biosystems Research Complex, Clemson University, 105 Collings St., Clemson, SC 29634-0973 USA

**Keywords:** Hydrophobic interaction chromatography, Size exclusion chromatography, C-CP fiber spin-down tips, Extracellular vesicles, Human embryonic kidney (HEK) cells

## Abstract

Human embryonic kidney (HEK) cell lines are used in the production of diverse biopharmaceuticals, including extracellular vesicles (EVs), due to their scalability and human biocompatibility. EVs are nanosized (30–200 nm), carriers of biomolecules that participate in cellular communication and can deliver therapeutic cargo in a targeted manner. Their use as drug vectors requires high-purity isolates; however, achieving such purity is challenging due to complex host matrices. Size exclusion chromatography (SEC) affects separations based on hydrodynamic radii and is a common isolation method due to its downstream applicability; however, it struggles to yield high-purity isolates. Polyester (PET) capillary-channeled polymer (C-CP) fiber spin-down tips are a novel EV separation platform employing hydrophobic interaction chromatography (HIC) to isolate high-purity EVs. Samples are loaded under high ionic strength conditions, where hydrophobic species adsorb to the fibers, and polar species pass through unretained. Analytes are then eluted in order of increasing hydrophobicity using an inverse salt gradient and adding small amounts of organic solvent at each step. A practical comparison of a commercial SEC column and PET C-CP spin-down tip EV eluates is conducted here via quantitative absorbance response curves, protein assays, nanoflow cytometry, and transmission electron microscopy. PET C-CP fiber platforms isolated 4.3 × 10^11^ particles mL^−1^ of HEK supernatant and yielded purities of 3.1 × 10^10^ particles µg^−1^ protein, a tenfold improvement for both metrics versus the SEC methodology, demonstrating a rapid, high-purity alternative to that commercial method.

**Supplementary Information:**

The online version contains supplementary material available at https://doi.org/10.1007/s00216-026-06653-5.

## Introduction

Extracellular vesicles (EVs) are nanosized (30–200 nm), membrane-bound vesicles that play an integral role in cell-to-cell communication and as biological delivery vehicles [1]. EVs are secreted from all cell types and are of interest for therapeutic vector applications due to their lack of immunogenicity and ability to transport a variety of cargo in a targeted manner to tissues throughout the body and across the blood–brain barrier [2, 3]. EVs are found in diverse matrices, ranging from cell culture supernatant and plant apoplastic wash to mammalian milk, where they transport biomarkers reflective of their host cell of origin; these contents make them excellent candidates for disease diagnostics [4–6]. Production of cell culture-derived EVs is commonplace in both industrial and academic settings and has been effectively used to produce biopharmaceuticals due to its scalability, capacity for transfection, and human biocompatibility [7]. Human embryonic kidney (HEK 293) cell lines are capable of rapid reproduction and have emerged as a promising host for EV production. They are currently utilized in the large-scale production of EVs due to the scalability of suspension growth platforms and have demonstrated high process reproducibility [7, 8]. As with other matrices, an obstacle in isolating EVs from HEK 293 supernatant is the complexity of the matrix itself, which includes media additives, proteins, cell debris, amino acids, salts/sugars, and often other bio-nanoparticles [7].

The use of EVs as biotherapeutics requires dosing concentrations on the order of 10^6^–10^11^ particles mL^−1^ [2, 9]. A crucial metric here is the purity of the product, that is, the ratio of particles to background protein content. The current purity target of 3.0 × 10^10^ particles µg^−1^ of latent protein has been established for biofluid-derived EVs [10], though this value is not mandated by any regulatory agencies. While useful as a benchmark, this purity standard is limited by the isolation methods and matrices used for validation, which are relevant when considering the proteinaceous nature of cell culture supernatant, mammalian milk, etc. Currently, there is no universal EV isolation method that is ideal for every matrix; therefore, isolation methods are commonly selected based on the specific downstream applications. Methods such as ultracentrifugation (UC), various forms of tangential field flow filtration (TFF), polymer precipitation, and several other forms of precipitation or filtration are commonly employed, each possessing strengths and challenges when it comes to EV isolation [11].

The most commonly applied of the EV isolation methods, UC employs high centrifugal forces to isolate matrix species based on density, with denser matrix-related species being sedimented first, followed by less dense species as the centrifugation speed increases. While this method is user-friendly, major drawbacks include long isolation times (2–24 h), equipment costs ranging from thousands to hundreds of thousands of dollars, the inevitable sedimentation of EVs with matrix species of similar densities (i.e., high-density lipoproteins (HDL)), and bias toward larger EVs [12, 13]. TFF and its variations, including asymmetric flow field-flow fractionation (AF4), affect isolation based on size as solvent is flowed tangentially across a membrane comprised of specified pore sizes; TFF is best suited for bulk (preparative) separations, as small sample volumes are heavily impacted by large dilution factors; however, co-isolation of matrix-related species of similar size poses purity issues [14–16]. Polymer precipitation kits are most ideal for isolating EVs from small sample pools, as they do not result in significant dilution; however, this method fails to yield high-purity products as EVs co-precipitate with the polymer used for dehydration [17, 18].

Finally, size exclusion chromatography (SEC) is a common isolation method that can be used as a stand-alone technique or integrated into existing workflows such as in multidimensional chromatography [19–21]. SEC separates analytes based on their hydrodynamic radius [22, 23]. Previously used for the separation of macromolecules, including proteins, peptides, and DNA, gravity-fed SEC columns have been successfully adapted for the EV arena [22, 23]. SEC columns employ a porous stationary phase of resin beads containing a network of pores of varying size. The diameter of the largest pores defines the exclusion limit, ideally 10 × the diameter of the analyte of interest [22, 23]. Species larger than the pore size exclusion limit (EVs in this case) will have minimal interactions with the stationary phase and will elute first, while smaller species diffuse within the porous stationary phase, increasing their retention time [23, 24]. Separations of 0.5 to 1 mL samples are possible in about 20 min, with decreased protein coelution in comparison to other methods, including UC, though purity remains a concern [24–27]. SEC columns have yielded EV particle counts of ~ 10^10^–10^11^ particles mL^−1^ and eluates with purities of ~ 10^8^–10^9^ particles µg^−1^ protein [20, 25, 26], below the target purity value. Dilution factors strongly impact isolations with small sample input volumes and matrices that are not EV-rich [24, 25, 27, 28]. Long process times limit throughput, as the porous stationary phase must be thoroughly regenerated and re-equilibrated with tens of column volumes of solvent between sequential separations, with each individual separation resulting in separation times of ~ 40 min [24, 25, 29, 30].

A novel EV separation platform, developed by Marcus et al., provides an alternative to conventional methods, decreasing isolation times and costs while maintaining purities and particle yields [31]. Polyester (PET) capillary-channeled polymer (C-CP) fibers are packed into polyether ether ketone (PEEK) capillaries for the isolation of a range of bio-nanoparticles, including EVs [4–6, 32–36]. Microbore columns are 0.76 mm i.d. × 30 cm in length and assembled at a cost of less than $5 a column, while smaller columns for spin-down tip applications are assembled with fluorinated ethylene-propylene (FEP) capillaries (0.8 mm i.d. × 1 cm in length) and cost < $0.50 per tip. When packed into the polymeric tubes, trilobal PET fibers interdigitate to create channels ranging from 1 to 4 µm in diameter, serving as a hydrophobic stationary phase. Using a hydrophobic interaction chromatography (HIC) solvent suite, EVs are isolated based on their hydrophobicity (relative to other matrix-related species) [36]. First, the sample is loaded onto the columns or spin-down tips under high ionic strength conditions, whereby matrix-related salts, sugars, and ionic compounds pass through unretained. Meanwhile, more hydrophobic species, including proteins and EVs, are adsorbed to the fiber surface. Proteins/lipoproteins are eluted by decreasing the ionic strength and adding a small amount of organic solvent. Finally, EVs are eluted by further decreasing the ionic strength and increasing the organic solvent content [36]. This rapid separation method allows for the tailoring of the organic elution solvent to meet specific application needs, thereby streamlining workflows. Acetonitrile (ACN) is most often used for the immediate evaporation and characterization of eluates, leaving EVs in 1 × PBS, while glycerol [6] and Tween-20 [37] are best suited for long-term storage before analysis.

This stationary phase is highly effective in producing EVs of high purity that are morphologically intact, with isolate particle counts on the order of  > 10^10^ particles mL^−1^ with purities of  > 3.0 × 10^10^ particles µg^−1^ protein from a plethora of matrices, including urine, plasma, serum, breast milk, bovine milk, cell culture, and plant apoplastic wash [4–6, 34–37]. PET C-CP fiber platforms are user-friendly and affect isolations in less than 10 min with high-throughput and purity at low costs. However, further comprehensive comparisons between PET C-CP spin-down tips and the more common methods for isolating EVs are necessary to evaluate the practical performance of spin-down tips. Here, a quantitative comparison of PET C-CP spin-down tips and commercial gravity-fed SEC columns was conducted through characterization of HEK 293 cell-derived EVs [22]. Eluates from both separation methods were subjected to a quantitative EV characterization suite, which included particle concentration determination via optical absorbance response curves generated using an EV reference material, and purity metrics using quantitative protein assays. Immunoconfirmation was performed using nanoflow cytometry, and vesicle morphology was examined via transmission electron microscopy (TEM). This quantitative comparison of EV eluates demonstrated that PET C-CP spin-down tips are an effective and inexpensive alternative to commercial isolation methods in the delivery of high-quality EVs for downstream applications.

## Methods

### Instrumentation

A VWR symphony 4417 tabletop centrifuge (Radnor, PA, USA) was used to effect the isolation of EVs via PET C-CP fiber spin-down tips. The NanoFCM Nanoanalyzer (nanoflow cytometer) (Nottingham, Nottinghamshire, UK) provided data regarding EV size distribution estimations and immunofluorescence confirmation of isolated EVs. An Agilent BioTek Synergy LX Multi-Mode plate reader (Santa Clara, CA, USA) was implemented for protein assay quantification detection. A JEOL 2100PLUS transmission electron microscope housed in the University of Georgia, Athens’ Georgia Electron Microscopy core facility was used for TEM imaging. A Thermo Fisher Scientific Vanquish Flex system high performance liquid chromatography (HPLC) instrument (Sunnyvale, CA, USA) equipped with a UV–Vis photodiode array detector (diode array detector FG) with a 13 µL biocompatible flow cell, autosampler (split sampler F), and quaternary pump (Quat. Pump F) was used to perform the optical absorbance-based EV response curves at 216 nm in the column bypass mode. The system was controlled using Chromeleon 7.3.2 software.

### Chemicals and reagents

Deionized (DI) water from an Elga PURELAB flex water purification system (18.2 MΩ cm^−1^) (Veolia Water Technologies, High Wycombe, England) was used for all solvents. A 10 × phosphate-buffered saline (PBS) solution was purchased from Thermo Fisher Scientific (Waltham, MA, USA) and diluted to 1 × for use in mobile phases and dilutions after being adjusted to pH 7.0. HPLC-grade ammonium sulfate (AMS) and acetonitrile (ACN) were purchased from VWR (Solon, OH, USA). PET-Y polyester fibers were purchased from Universal Fibers, Inc. (Bristol, VA). Fluorinated ethylene polypropylene (FEP) tubing (Cole Parmer, Vernon Hills, IL, USA) was packed with PET C-CP fibers to construct spin-down tip columns [4, 6, 37]. Pipette tips (1 mL and 200 µL) were used to assemble PET C-CP spin-down tips, and 15-mL conical tubes were used as sample reservoirs (VWR, Radnor, PA, USA). Calibration of the NanoFCM, Inc. (Nottingham, Nottinghamshire, UK), nanoanalyzer instrument was performed according to manufacturer specifications using 0.25-µm fluorescent silica microspheres (QC Beads) with a particle count of 2.17 × 10^10^ particles mL^−1^, and a silica nanosphere standard with four distinct size populations (68, 91,113, and 155 nm) in 1:99 dilutions with DI. Both calibration standards were purchased from NanoFCM. Immunoconfirmation was conducted with anti-CD81 and anti-CD9 fluorescent antibodies purchased from Biotium (1:2000 final dilution) (Freemont, CA, USA). MemGlow (Cytoskeleton Inc., Denver, CO, USA), a lipophilic anchor membrane dye, intercalates with the vesicle membrane to fluorescently label vesicles in a generic manner (1:4000 final dilution). HEK 293 cell culture supernatant, obtained through a collaboration with Dr. Sarah Harcum’s laboratory at Clemson University, was filtered through a 0.22-µm polyethersulfone (PES) syringe filter (FroggaBio, Toronto, Canada). HEK 293-derived EV lyophilized standards (3.7 × 10^11^ particles mL^−1^) from HansaBioMed (Tallinn, Estonia) were used to construct absorbance response curves for EV quantification. A microBCA assay kit was purchased from Thermo Fisher Scientific (Waltham, MA, USA) for protein quantification. TEM imaging required carbon-coated copper grids, 1% uranyl acetate, and 2% paraformaldehyde (16% stock concentration), purchased from Electron Microscopy Sciences (Hatfield, PA, USA). The 4B Size Exclusion Chromatography Sepharose Columns were provided by Everest Biolabs (Waltham, MA, USA).

### HIC PET C-CP fiber spin-down tip method

The PET C-CP fiber spin-down tips were constructed according to previously established protocols [4, 6, 37]. Here, six rotations (396 fibers) of PET-Y (trilobal) fibers were spooled and heat-shrunk using hot water [4]. Heat-shrunk fibers were fed through 30 cm of FEP with an inner diameter of 0.8 mm and pulled out of the tubing to create a 0.5 cm space where a 200-µL micropipette tip was inserted with a small amount of household adhesive. The packed capillary was cut to create a 1-cm fiber column with an interstitial fraction of ~ 0.6 with an ~ 3 µL bed volume. The 200-µL pipette tip with the attached column was nested in a 1-mL pipette tip, which served as the spin-down tip.

A cap from a 15-mL conical tube was customized to hold the tip assembly by drilling a hole through its center and nesting a microcentrifuge tube with the bottom cut off. The spin-down tip was then placed in the customized cap and screwed onto the 15-mL conical tube for EV isolation using a tabletop centrifuge. Biocompatible polymer microcentrifuge tubes with the lids removed were inserted into 15-mL conical tubes to serve as fraction collectors. HEK 293 supernatant and 4M AMS were combined in equal parts (1.2 mL each) in a 15-mL conical tube and inverted to mix thoroughly. The sample mixture was then applied to the PET C-CP fiber tips for EV isolation as previously described [4]. First, 200 µL of the sample loading mixture was added to the spin-down tip and centrifuged for 2 min at 300 × g, where the ionic compounds and small particulates passed through unretained, while hydrophobic species adsorbed to the fiber surfaces. Proteins were eluted by adding 200 µL of 1M AMS and 10% ACN to the tip reservoir and centrifuging for 2 min at 300 × g. Finally, for EV fraction collection, a microcentrifuge tube with no lid was inserted into a 15-mL conical tube before the addition of 200 µL of 35% ACN in 1 × PBS. Important here is the fact that the original 100 µL sample aliquot was processed to deliver the final EV product, leaving the sample in a 100 µL PBS matrix, i.e., without dilution. After the final elution centrifugation step (2 min at 300 × g), fractions containing EVs were pooled and stored uncapped at 5 °C overnight for ACN evaporation.

### Gravity-fed size exclusion columns

The Everest Apex (Waltham, MA, USA) 4B SEC gravity-fed columns’ stationary phase consists of 4% cross-linked agarose beads with an exclusion limit of 35 nm. The manufacturer states these columns are optimized for high-purity isolations of EVs from biofluids. The columns were used for EV separations after being equilibrated according to the manufacturer’s instructions [21]. Before each isolation, the columns were equilibrated by passage of 10 mL of 1 × PBS buffer, a 10-min process. The isolation procedure began with 500 µL of the sample loaded onto the packed bed. Once the column stopped dripping, indicating the sample was completely loaded onto the column (~ 45 s), 2 mL of PBS was added and the eluate discarded. Once the column stopped dripping for ~ 1 min, 500 µL aliquots of PBS were added to elute EVs. For each aliquot, eluate was collected in biocompatible microcentrifuge tubes until the column stopped dripping. For these experiments, three Apex 4B columns were run in parallel. Fractions 1, 2, and 3, which constitute the full EV population (according to the manufacturer), were pooled and stored at 5 °C for subsequent quantification. Columns were washed by flushing with 4 mL of 0.5 M NaOH and then requilibrated with 20 mL of 1 × PBS.

### Validation of vesicular structure

Maintaining and verifying the integrity of EV morphology post-isolation is imperative toward the validation of any separation platform. EV samples from both HIC and SEC separation methods were negatively stained using uranyl acetate, according to a previously established protocol with some adjustments [38]. Carbon-coated copper grids were incubated twice with 10 µL of sample isolates for 10 min, with excess sample wicked off between incubations using filter paper. The grids were then rinsed by dipping briefly in one drop of distilled water before wicking dry. Grids were placed upside down on a drop of 2% paraformaldehyde for 5 min and then washed by dipping in three drops of 50 mM phosphate buffer, allowing the grid to sit in the last drop for 2 min. Using filter paper, the grid was wicked dry and allowed to rest for 15 s in a 1% uranyl acetate solution before being wicked dry again. Negatively stained samples were incubated in a dehumidifier for ~ 30 min and imaged via a JEOL 2100PLUS microscope at 120 kV.

### Size distribution, particle count, and immunoconfirmation

The NanoFCM Nanoanalyzer utilizes two lasers, one red (680 nm) and one blue (488 nm), with three channels/bandpass filters for side scatter and fluorescent detection. The instrument provides data regarding the size profile of the sample, concentration estimations, and simultaneous detection of two unique fluorescent labels. The three channels include side scattering (bandpass filter 488/10), FITC (bandpass filter 525/40 nm), and PE-Cy5 (bandpass filter 670/30 nm). All solvents used with the instrument are filtered with a 0.22-µm polytetrafluoroethylene (PTFE) hydrophilic syringe filter (Thermo Fisher Scientific, MA, USA). The instrument is calibrated according to manufacturer instructions using 1:99 dilutions in DI water of 0.25 µm fluorescent silica microspheres (QC Beads) with a particle count of 2.17 × 10^10^ particles mL^−1^ for concentration calibration. A second 1:99 dilution in DI water of a silica nanosphere cocktail (S16M-Exo) with 68, 91, 113, and 155 nm beads is used for size calibration. Once calibrated, the instrument is used to detect unlabeled and fluorescently labeled species.

In this case, two distinct fluorescent labels were selected for detection: a lipophilic anchor membrane dye that intercalates with the vesicle lipid bilayer and an anti-CD81 and anti-CD9 fluorescent antibody (Ab) cocktail that targets tetraspanins within the EV membrane. Here, instrumentation blanks of neat 1 × PBS solution, EV isolates that were not fluorescently labeled, and fluorescent dyes in 1 × PBS were used. The fluorogenic membrane probe (1:4000 final dilution, MemGlow™) was detected at 680 nm, and the anti-CD81/anti-CD9 cocktail (1:2000 final dilution, Biotium) was detected at 516 nm. Before incubation with fluorescent labels, ACN in the PET C-CP spin-down tip EV fractions was evaporated overnight at 5 °C. The fractions were then diluted to ~ 2.0 × 10⁸ particles mL⁻^1^ with 1 × PBS. The samples were incubated for 2 h in a water bath (37 °C) protected from light, and then gently vortexed and introduced to the NanoFCM Nanoanalyzer according to the manufacturer’s instructions. The operation of the instrument and data analysis were controlled via the NF Profession 2.3 software.

### Particle quantification

An absorbance-based response curve was constructed for particle quantification of EV eluates using serial dilutions of lyophilized HEK 293 EV standard with a particle count of 3.7 × 10^11^ particles mL^−1^ from HansaBioMed (Tallinn, Estonia) [36]. A Thermofisher Vanquish Flex Horizon (Waltham, MA, USA) with an autosampler and UV detector at 216 nm was used for quantification. In this case, 2-µL injections of EV standard and the series of serial dilutions were introduced into 1 × PBS at a flow rate of 0.5 mL min^−1^. The response curve spanned particle concentrations of 2.31 × 10^10^–3.70 × 10^11^ particles mL^−1^ with linearity of *R*^2^ = 0.98 from triplicate injections. After the ACN was evaporated from PET C-CP spin-down tip fractions overnight and diluted to the same fixed volume, EV isolates from both methods were injected in the same manner as the standards.

### Protein content and purity

Total protein content from HEK293 supernatant (diluted 1:50 with 1 × PBS) and fractions from both SEC and PET C-CP spin-down tips were assessed using a microBCA protein assay based on the equipment manufacturer’s instructions. EVs eluted from the PET C-CP spin-down tips were evaporated overnight at 5 °C and diluted to the same fixed volume, leaving the EVs in 1 × PBS. SEC fractions remained in 1 × PBS post-isolation. The response curve for the assay was constructed according to the manufacturer’s instructions using a primary bovine serum albumin (BSA) standard from Thermo Fisher Scientific (Waltham, MA, USA), spanning a concentration range of 0.5 to 200 µg mL^−1^ as shown in Supplementary Fig. 1. The microBCA assay utilized 150 µL of standard/sample and 150 µL of microBCA working reagent prepared according to the manufacturer’s protocol; each sample and standard was loaded in triplicate. Detection occurred at 562 nm via the microplate reader after mixing for 30 s and a 2-h benchtop incubation period at 22 °C. This value is lower than the manufacturer’s suggestion of 37 °C to further ensure the vesicles’ integrity. The retention of structural integrity has been demonstrated in previous experiments where a microBCA assay of RIPA-lysed and intact EV eluates yields significant differences in absorbance responses [5]. Purity of EV isolates (EVs µg^−1^ protein) was determined using total protein assay data and particle count values obtained from response curves.

## Results and discussion

The development of novel isolation methods is imperative to the advancement of the downstream applications of EVs. As the matrices EVs are sourced from continue to diversify, so does the need for versatile and robust isolation platforms that deliver high-quality products. In comparing PET C-CP spin-down tips and SEC for the isolation of EVs using quantitative methods and characterization techniques, a comprehensive validation of PET C-CP spin-down tips is achieved.

### Validation of vesicular structure

**Fig. 1:**
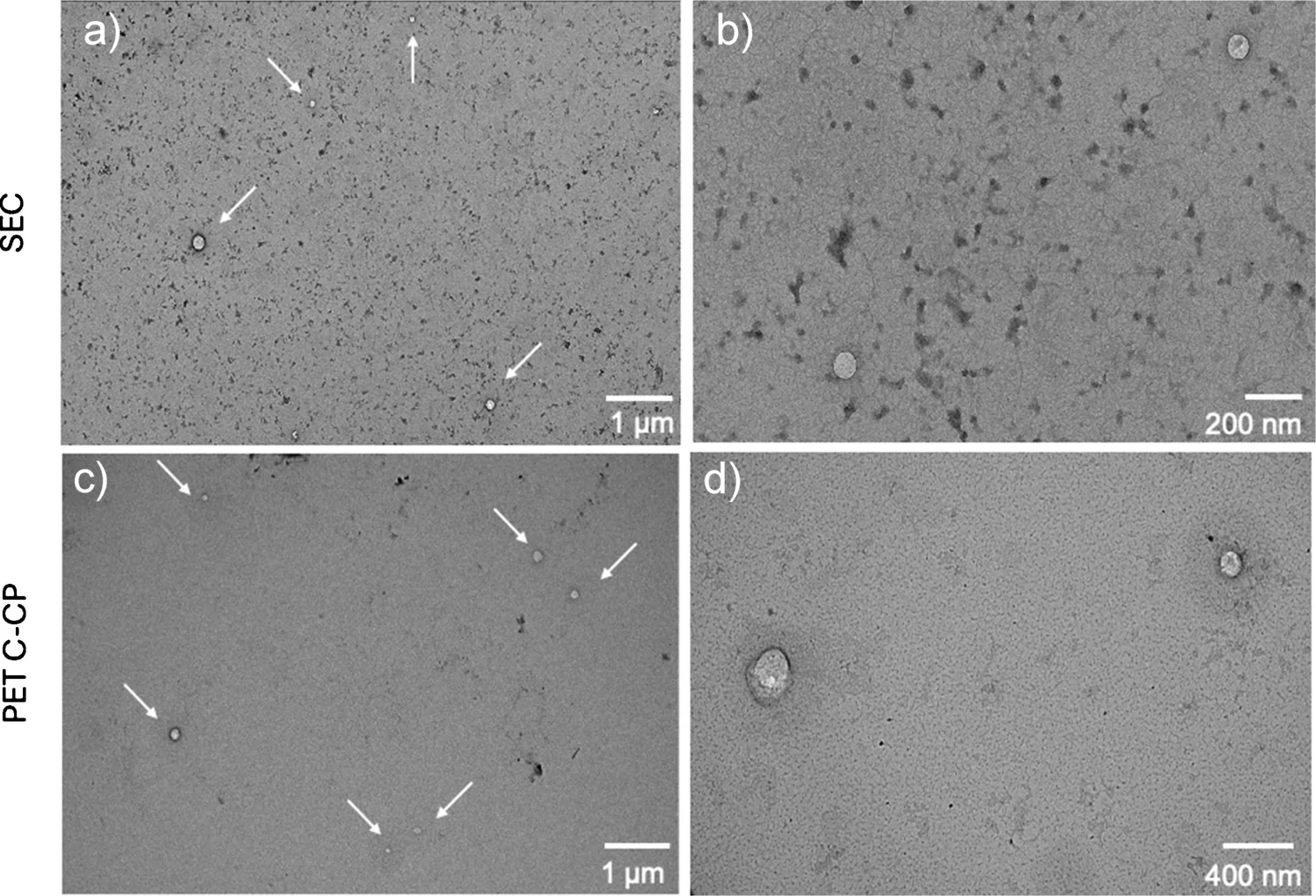
TEM micrographs of HEK 293-derived EVs with negatively stained lipid bilayers isolated via **a** SEC with multiple EVs in the wide field view (1 µm) within the size range of 30–200 nm, **b** close field view (200 nm) of two EVs from the previous micrograph, **c** EV eluates from PET C-CP spin-down tips with a wide view (1 µm) of six EVs in frame within the size range of 30–200 nm, and **d** close field view (400 nm) of two EVs

A primary measure of success in isolating EVs is the retention of vesicle morphology. Currently, the “gold standard” method for verifying vesicle morphology is the imaging of negatively stained EVs via TEM. An effective isolation method yields EVs with spherical morphology and an intact phospholipid bilayer, indicating that the EVs were undamaged by the employed isolation method. After uranyl acetate negative staining of the EVs, a 3-D structure is indicated by the dark shadow outside the membrane, demonstrating the depth of the vesicle. Figure 1 presents TEM micrographs of the eluates from the two methods, taken on broad and focused field scales. The intent here is to give a qualitative reflection of the cleanliness of the fractions without preconcentration/alteration, as well as the retention of the vesicular structure for the methods, supplementing the other diagnostic methods. Figure 1a and b demonstrates a uranyl acetate-stained EV population from an SEC separation. In Fig. 1a, the ~ 5 × 10 µm micrograph exhibits a section of the TEM grid with multiple vesicles in frame, highlighted by white arrows. Spherical vesicles possessing intact phospholipid bilayers are clearly seen, exhibiting the depth expected from negative staining. The sizes of the vesicles are within the range characteristic of EVs, with the two vesicles highlighted in Fig. 1b being ~ 100 nm in diameter. The TEM images of the EVs isolated using PET C-CP spin-down tips are shown in Fig. 1c and d; these vesicles exhibit a clearly intact phospholipid bilayer and a spherical shape, as well as having a 3-D structure. In Fig. 1c, the wide-field view shows a plane of the grid with a 1-µm scale bar where several vesicles of various sizes are indicated by white arrows, as well as some uranyl acetate stain and/or matrix-related aggregates toward the right side of the micrograph. The final image, Fig. 1d, shows a close-up micrograph of two vesicles on a 400-nm scale, providing a clearer demonstration of their intact morphology, including the negatively stained lipid bilayer and spherical shape. The vesicles are ~ 100 nm, with smaller vesicles visible in the previous micrograph. In all four micrographs, small aggregates are visible in the background, which are attributed to aggregated matrix-related species stained with uranyl acetate, and/or aggregates of the uranyl acetate itself [39]. Notably, there are considerably fewer background species in the PET C-CP spin-down tip images. Clearly, both methods yielded vesicles with morphological features characteristic of EVs. These observations contribute to the validation of the high-purity and non-denaturing nature of the PET C-CP spin-down tip method.

### Particle sizing and immunoconfirmation via nanoflow cytometry

**Fig. 2:**
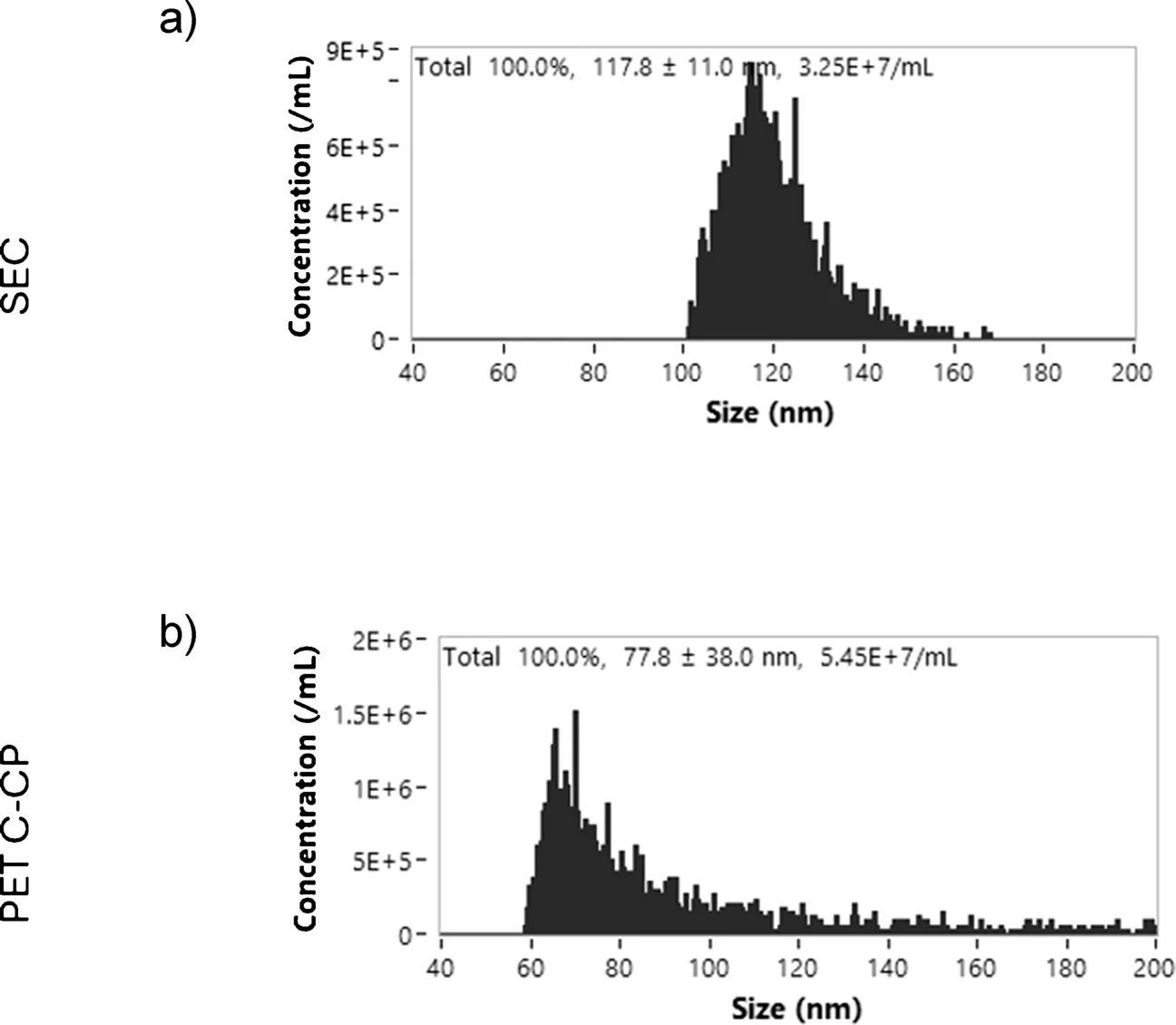
NanoFCM size distribution histograms of EVs from **a** SEC eluates with particles in the size range of ~ 100–160 nm and **b** PET-Y C-CP spin-down tip eluates in the size range of ~ 60–160 nm

The NanoFCM Nanoanalyzer contributes to EV characterization by providing size profiles and immunofluorescence detection. The histograms in Fig. 2a and b depict the size distributions of EV eluates from the SEC and PET C-CP spin-down tip isolations, respectively. The size distribution plot from SEC eluates (Fig. 2a) shows EVs with an average size of 117.8 ± 11 nm, corresponding to a 2.2% relative standard deviation (RSD) (*n* = 3). EVs with an average size of 77.8 ± 38 nm from the PET C-CP spin-down tips are shown in Fig. 2b with a 3.4% RSD (*n* = 3). The difference in the size distributions from SEC and HIC PET spin-down tips is not surprising when considering the distinct stationary phases and the physio/chemical separation pathways. The SEC column manufacturer recommends the collection of the first three fractions for the highest purity isolates. By definition, fractions collected later in the isolation scheme would likely include smaller matrix-related species, and so the size distribution of the first three fractions would be biased to larger particles. Fractions collected later in the SEC isolation scheme often contain EVs co-eluted with large protein aggregates and/or exomeres [22, 23, 40]. PET C-CP tips isolate EVs based on their hydrophobicity relative to other matrix species, where smaller EVs, due to their increased surface area to volume ratio, would be expected to experience greater hydrophobic interactions on the stationary phase than larger EVs. Therefore, it is reasonable to suggest that HIC tips preferentially isolate smaller EVs, though a dedicated study would be necessary to confirm. The isolation of a smaller-sized population of EVs via PET C-CP tips holds potential for therapeutic spaces, as small EVs are reportedly internalized by cells primarily distributed in the lungs, liver, and spleen, though biodistribution tracking remains a challenge [41–43]. To this end, a very recent report from this laboratory has demonstrated the ability to perform sub-population differentiation via HIC chromatography on the C-CP fiber column platform [44].

**Fig. 3:**
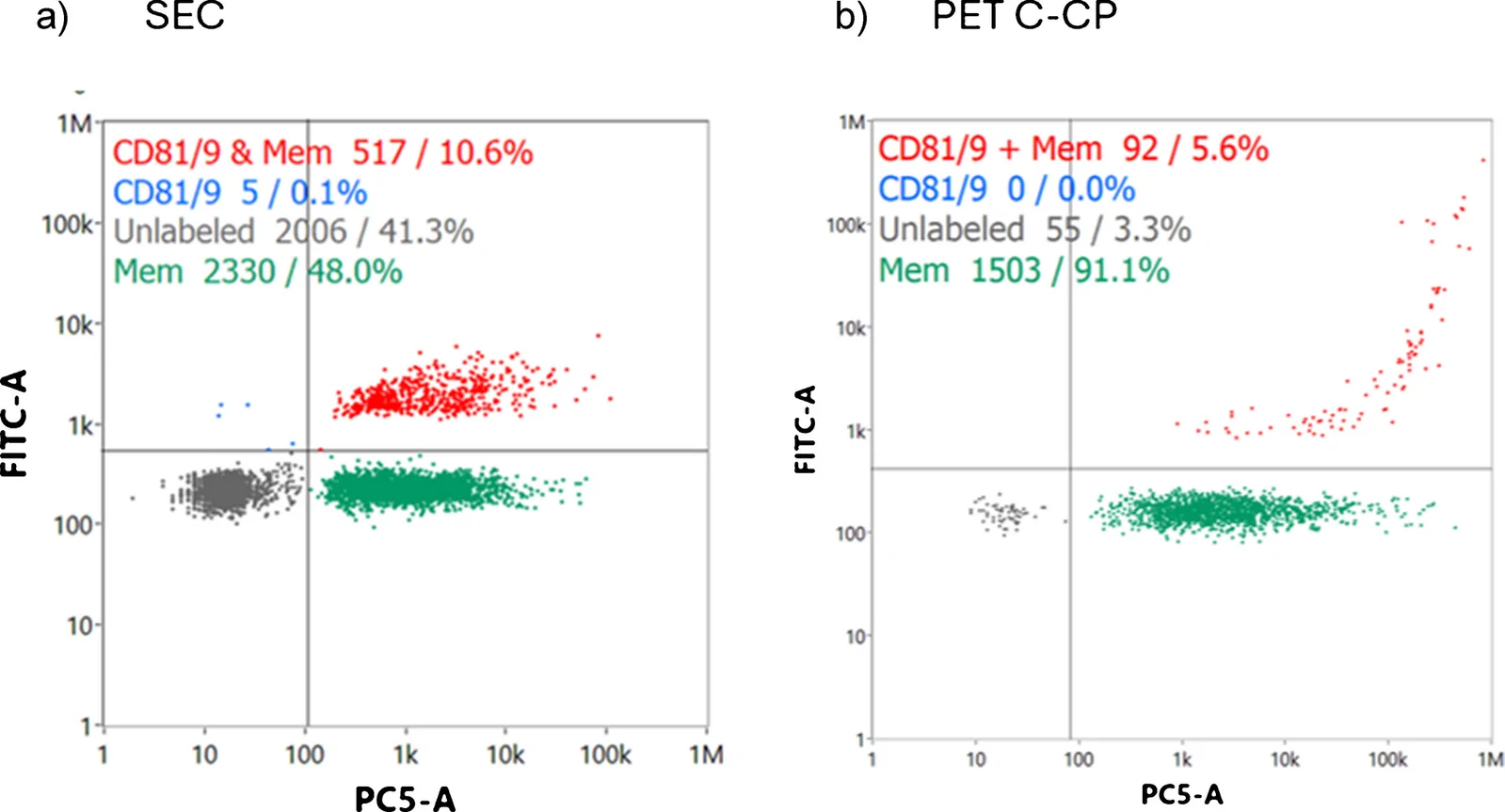
NanoFCM quad plots representative of post-isolation EVs fluorescently labeled with anti-CD81, anti-CD9, and membrane vesicle dye: **a** Quad plot of fluorescently labeled EVs isolated via SEC and **b** quad plot of fluorescently labeled EVs isolated via PET C-CP spin-down tips

Immunoconfirmation of EVs was performed using two fluorescent labels, each targeting a unique aspect of EV morphology: the lipophilic membrane dye for vesicle membrane confirmation and a cocktail of anti-CD81 and anti-CD9 Abs targeting tetraspanin protein markers within the EV membranes. EV eluates were adjusted to ~ 10^7^ particles mL^−1^ before fluorescent labelling and incubation, as recommended by the manufacturer. Figure 3a and b depicts representative quad plots of EVs separated via SEC and PET C-CP spin-down tips. Each quad plot represents the median result of triplicate analysis from each separation, with standard deviations relating to labeling efficiency between the replicates being 0.5% and 0.3% for the SEC and PET C-CP plots, respectively. Important in the interpretation of these plots is the required correlation of the target species’ fluorescent responses to individual side-scattering events. In these plots, data points in the bottom left quadrant reflect particles which scattered light, but had acquired no fluorescent tags. Responses in the top left quadrant reflect particles which exhibit side-scattering but only antibody labeling, while those in the bottom right quadrant are those that only exhibit membrane dye labeling. Finally, the top right quadrant reflects those particles which scattered laser light and had received both forms of labeling.

The quad plot presented in Fig. 3a is representative of the EV eluates from the SEC separations. The bottom left quadrant depicts particles detected through scattering with no fluorescent response, i.e., not labeled, representing 41.3% of the total registered events. The top left quadrant represents particles only labeled with anti-CD81/9. The lack of positive response (0.1%) is expected as tetraspanins are membrane-integrated biomarkers due to the low abundance of those proteins. That said, the higher value of the double-positive response of 10.6% in the top right quadrant reflects the very high affinity toward labeling by the vesicular dye, as it also receives the immunofluorescent label. The overall high labeling efficiency of the lipophilic membrane dye is observed in the bottom right quadrant, for those particles responding positively in the corresponding fluorescent channel, that did not acquire the antibody labels. Overall, a total of 58.6% of the particles exhibit a positive response for the vesicular dye.

The corresponding fluorescence assay for the EV eluates from PET C-CP spin-down tips is presented in Fig. 3b. In this case, the lower left quadrant demonstrates a small (3.3%) population of particles that registered a positive response only in the scattering channel. Comparable to the quad plot for the SEC-derived EVs, no particles registered a positive response in the top left quadrant corresponding to the fluorescent channel solely for anti-CD81/9 labeling. The high labeling efficiency of the vesicle membrane is observed in the bottom right quadrant, where 91.1% of particles registered a positive fluorescent response for the membrane dye only. Finally, in the top right quadrant, a positive response of 5.6% is registered for particles labeled with both the vesicle membrane dye and fluorescent anti-CD81/9 cocktail. Overall, 96.7% of the detected particles registered a positive response for vesicle membrane dye.

A 5% difference in the double-labeling efficiency is observed in the detection of positive events between the eluates of the SEC columns and the PET C-CP spin-down tips in the top right quadrants of Fig. 3a and b. The difference in positive response is not surprising, as it has been reported in literature that smaller-sized vesicles secreted via distinct pathways do not express these tetraspanins at all, and would therefore not be observed via nanoflow cytometry in the antibody fluorescence channel [45]. It is expected that the response from SEC EV eluates for CD Abs would be higher than that of the PET C-CP spin-down tips, as the average particle size of the eluates differs by 40%. Thus, in comparison to the smaller spin-down tip EVs, each particle would have a greater likelihood to yield fluorescent responses above the detection threshold [46].

Beyond the greater detection toward the dual labeling, there are fewer overall unlabeled events in the PET C-CP spin-down tip quad plots, indicating a decrease in the coelution of non-vesicle-related species in the final EV eluate. While these experiments served as confirmation of vesicle presence and the non-denaturing nature of PET C-CP spin-down tips toward tetraspanin markers, we acknowledge the possibility that remnant ACN in the EV fractions may be limiting the fluorescent labeling process [47]. To address solvent interference with fluorescent labeling, the use of 10 kDa filters for solvent exchange has been successfully integrated with other PET C-CP platform workflows. However, as the goal of this study was a quantitative comparison, solvent exchange was not used here to ensure no particles were lost during the solvent exchange process. The EV eluates from both SEC and PET C-CP spin-down tips demonstrated positive responses toward two unique fluorescent labels used to target unique aspects of EV morphology vital to downstream processes.

### Particle quantification and eluate purity

When assessing EV eluates from differing isolation methods, process yield and purity are critical considerations for any downstream applications. Here, after pooling EV fractions from each separation method, particles were quantified via an optical absorbance response curve constructed using the Thermo Fisher Scientific Vanquish Flex system in the column bypass mode. It should be emphasized that while the surface contents of EVs may absorb light at 216 nm, EVs themselves scatter the light, and their quantification here is based on the detection of that scattering [36]. Although an HEK EV reference material is used here to construct the response curve, there are no certified or standard reference materials that demonstrate levels of purity that align with the benchmark of 3.0 × 10^10^ particles µg^−1^ protein with absolute particle densities. Thus, the “standards” available are used for quantification as part of this characterization suite.

The response curve constructed with serial dilutions of the HEK 293 EV reference material is presented in Supplementary Fig. 2, showing the expected linearity with an *R*^2^ value of 0.98 (*n* = 3). Reported EV recoveries were normalized to reflect recoveries per mL from the primary HEK 293 supernatant, as the input volume processed for the separation methods differed. SEC yielded a value of 4.3 × 10^10^ particles mL^−1^ of HEK 293 supernatant with 13% RSD. The PET C-CP tips yielded 4.9 × 10^11^ particles mL^−1^ of HEK 293 supernatant with 3% RSD, improving upon recoveries demonstrated by SEC. Therefore, the total particles purified via PET C-CP tips and SEC separation methods were 4.9 × 10^11^ particles and 4.3 × 10^10^ particles, respectively. In both cases, the values are in general agreement with particle counts from the literature [40, 48].

**Fig. 4:**
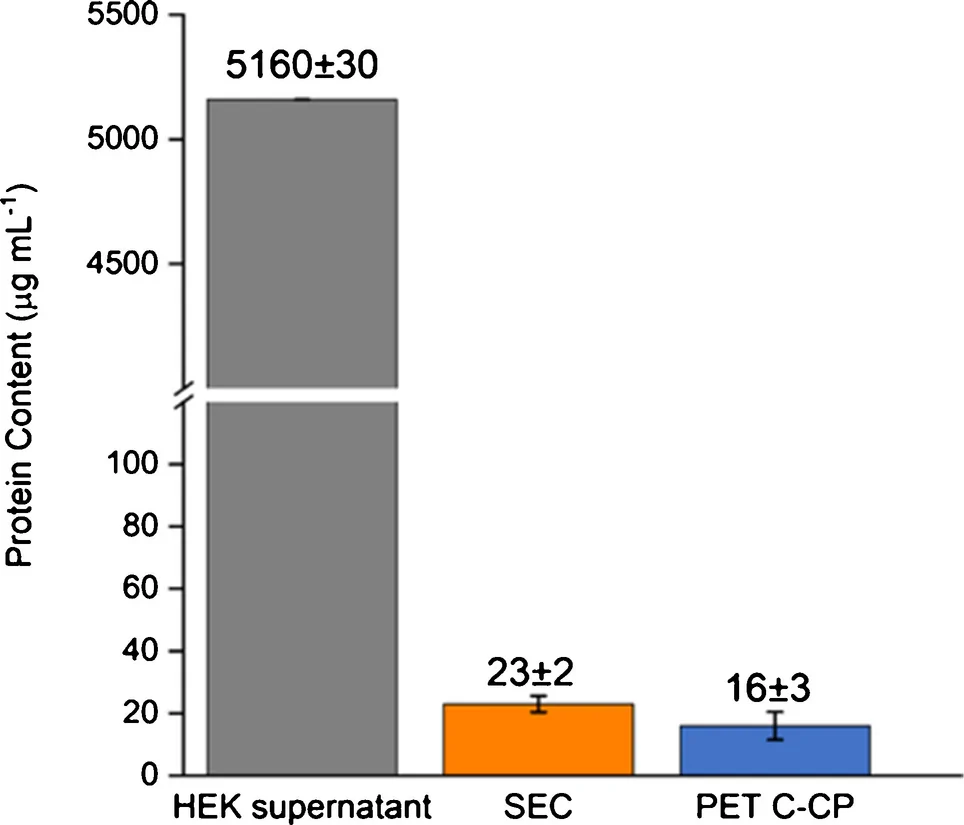
MicroBCA protein assay reflecting total protein content (µg mL^−1^) in HEK supernatant and both SEC and PET C-CP spin-down tip EV eluates

**Fig. 5:**
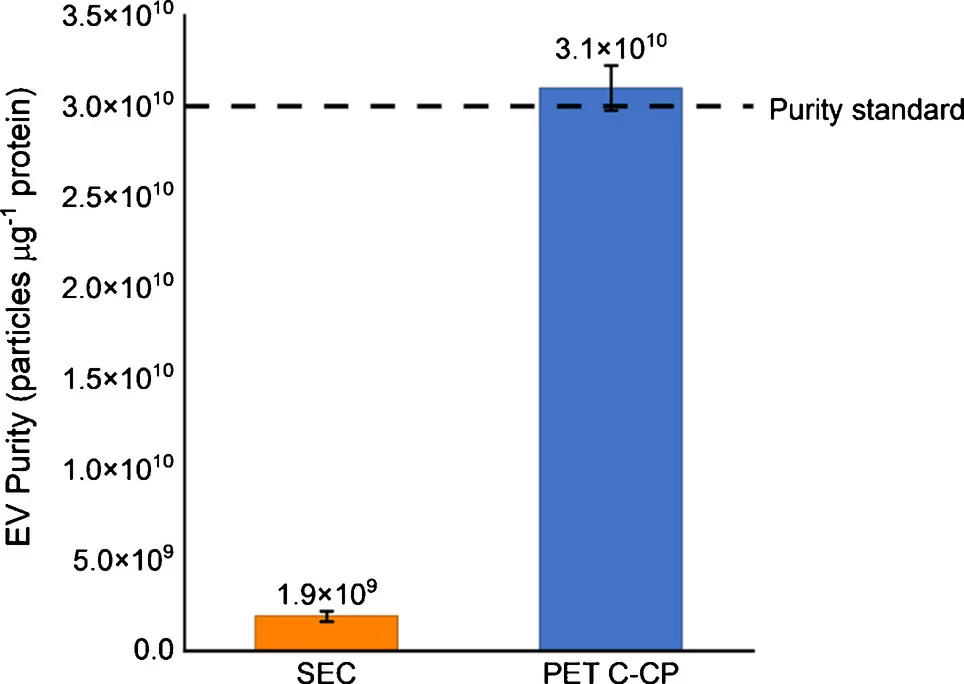
Comparison of EV eluate purity from SEC and PET C-CP isolation methods, with the dashed line designating the established purity value for biofluids

Eluant purity, a crucial metric relevant for downstream applications, was determined via a microBCA protein assay where total protein content in the EV fractions from both isolation methods was quantified. An important consideration when assessing total protein content is the selectivity of the microBCA assay reagents, as the working reagent will respond positively to EV membrane-related proteins, as well as free or fragmented proteins and amino acids in the matrix [49]. Marcus et al. have previously demonstrated that the microBCA reagent and incubation conditions alone are not sufficient to lyse the EV membranes [5]. In that case, by comparing microBCA responses of RIPA-lysed milk-derived EVs and intact EVs, the lysed EVs demonstrated approximately twice the response of the intact EVs, reflective of freed vesicular cargo [5]. Therefore, it is expected that the present microBCA results are reflective only of the free and membrane-associated proteins. Both separation methods produced EV eluates with significantly reduced protein content relative to the initial HEK supernatant sample, as demonstrated in Fig. 4. Before separation, the HEK293 supernatant demonstrated a protein concentration of 5160 ± 30 µg protein mL^−1^. The pooled EV fraction from the SEC columns contained 23 ± 2 µg mL^−1^ of protein, with the total protein content decreasing ~ 220-fold in comparison to the initial HEK 293 supernatant. PET C-CP fiber spin-down tip EV eluates demonstrated a ~ 320-fold decrease in protein content from the initial supernatant value, with a concentration of 16 ± 3 µg protein mL^−1^. As shown in Fig. 5, the gravity-fed SEC columns yielded EVs with a purity of 1.9 × 10^9^ particles µg^−1^ protein, in agreement with values reported in the literature [28, 50, 51]. PET C-CP spin-down tips yielded EVs with a purity of 3.1 × 10^10^ particles µg^−1^ protein, superior to the purity target of 3.0 × 10^10^ particles µg^−1^ protein, indicated by the dashed line [10]. The particle counts and purity of EV isolates obtained from HIC PET fiber spin-down tips are ~ 10 × higher than those of SEC eluates, quite impressive given the simplicity of the method. Notably, this purity value supersedes that of the HEK293 EV standard, which, while not explicitly stated, is calculated at ~ 3.7 × 10^9^ particles µg^−1^ protein. These metrics convey the PET C-CP spin-down tip method’s capacity to yield EV eluates that are morphologically intact, within the expected EV size range, and, most importantly, meet current purity standards.

## Conclusions

As new applications and demands for separation platforms that are capable of high-yield and purity isolation of EVs increase, it is crucial that separation platforms meet the performance metrics set by diverse end users. To address the need for an inexpensive yet robust and versatile EV separation method, Marcus et al. have developed the PET C-CP spin-down tip for rapid EV isolation and quantification [31]. This platform has proven to be user-friendly while delivering high throughput, high purity, and morphologically intact vesicles on very short time scales (10 min). Here, a quantitative comparison of PET C-CP spin-down tips and gravity-fed SEC columns was conducted. Table 1 demonstrates a comparison of the quantitative figures of merit for EV isolates from both SEC and PET C-CP spin-down methods. As demonstrated via TEM, SEC columns yielded morphologically intact vesicles within the expected EV size range. The non-denaturing nature of the PET C-CP spin-down tip method is also demonstrated through TEM micrographs, where EVs maintained their morphological properties. Interestingly, SEC isolated EVs having an average size of 117.8 ± 11 nm as determined by nanoflow cytometry scattering, while PET C-CP spin-down tips isolated a smaller size population with an average size of 77.8 ± 38 nm, a broader distribution. This difference in average EV size falls in line with the expectation that SEC would preferentially pass larger particles, while the fibers may preferentially capture smaller vesicles. Through nanoflow cytometry, immunodetection of vesicle membranes and tetraspanins integral to intercellular communication was possible, indicating both methods were successful in isolating EVs from the HEK 293 cell culture supernatant. As determined through quantitative absorbance curves, SEC yielded 4.3 × 10^10^ particles mL^−1^ of primary HEK supernatant with a purity of 1.9 × 10^9^ particles µg^−1^ protein in about 20 min. In comparison, the PET C-CP spin-down tips isolated 4.9 × 10^11^ particles mL^−1^ of HEK supernatant, an order of magnitude more than SEC, with a purity of 3.1 × 10^10^ particles µg^−1^ protein in 10 min. Through the quantitative characterization and comparison of EVs isolated via gravity-fed SEC and PET C-CP spin-down tips, PET C-CP spin-down tips demonstrated figures of merit that are on par with, and in some instances outperform those of SEC. Notable advantages of PET C-CP spin-down tips include low material costs (< $0.50 per tip), short time scales for isolation (< 10 min) and re-equilibration, high throughput, and superior purity to SEC. While PET C-CP fiber platforms have repeatedly demonstrated their efficacy in isolation of EVs from complex matrices, the next logical step is to validate the preservation of bioactivity. With this goal in mind, studies using HEK 293-derived EVs isolated via PET C-CP platforms for cellular uptake studies are underway.

**Table 1:** Figures of merit for the comparison of EV eluates from PET C-CP spin-down tips and commercialized SEC columns

	Particle count (particles mL^−1^ HEK supernatant)	Avg. size (nm)	Purity (particles µg^−1^ protein)	Time (min)	Cost ($)
PET C-CP	4.9 × 10^11^	77.8 ± 38	3.1 × 10^10^	10	< 0.50
SEC	4.3 × 10^10^	117.8 ± 11	1.9 × 10^9^	20	50

## Supplementary Information

Below is the link to the electronic supplementary material.

Supplementary file1 (DOCX 75.8 KB)

## Data Availability

Data employed in support of this manuscript are housed in the research laboratory and may be obtained upon written request.
