# Supplementary material for: Comparison of polyester (PET) capillary-channeled polymer (C-CP) fiber spin-down tips and size exclusion chromatography (SEC) for the isolation of HEK 293-derived extracellular vesicles (EVs)
Source: Anal Bioanal Chem. 2026 Jul 9;418(19):6307–18. doi: 10.1007/s00216-026-06653-5 (PMC13585934; doi:10.1007/s00216-026-06653-5)
Supplement: Supplementary file 1 [file 216_2026_6653_MOESM1_ESM.docx]

**Supplementary Information**

**for**

**Comparison of polyester (PET) capillary-channeled polymer (C-CP) fiber spin-down tips and size exclusion chromatography (SEC) for the isolation of HEK 293 derived extracellular vesicles (EVs)**

Carolina Mata, Christopher D. Topper, and R. Kenneth Marcus*

*Department of Chemistry, Biosystems Research Complex, Clemson University, Clemson, SC 29634-0973, USA*

*Corresponding Author: Prof. R. Kenneth Marcus, Department of Chemistry, Biosystems Research Complex, 105 Collings St., Clemson University, Clemson, SC 29634, USA; ORCID # 0000-0003-4276-5805, [marcusr@clemson.edu](mailto:marcusr@clemson.edu)

Sup. Fig. 1: BSA standard curve for MicroBCA protein assay.

Sup. Fig. 2: HEK293-derived EV standard curve for quantification of SEC and PET C-CP spin-down tip EV eluates.
